# Knee-clicks and visual traits indicate fighting ability in eland antelopes: multiple messages and back-up signals

**DOI:** 10.1186/1741-7007-6-47

**Published:** 2008-11-05

**Authors:** Jakob Bro-Jørgensen, Torben Dabelsteen

**Affiliations:** 1 Institute of Zoology, Zoological Society of London, London NW1 4RY, UK and Department of Biological and Environmental Science, University of Jyväskylä, Finland; 2 Animal Behaviour Group, Department of Biology, University of Copenhagen, Denmark

## Abstract

**Background:**

Given the costs of signalling, why do males often advertise their fighting ability to rivals using several signals rather than just one? Multiple signalling theories have developed largely in studies of sexual signals, and less is known about their applicability to intra-sexual communication. We here investigate the evolutionary basis for the intricate agonistic signalling system in eland antelopes, paying particular attention to the evolutionary phenomenon of loud knee-clicking.

**Results:**

A principal components analysis separated seven male traits into three groups. The dominant frequency of the knee-clicking sound honestly indicated body size, a main determinant of fighting ability. In contrast, the dewlap size increased with estimated age rather than body size, suggesting that, by magnifying the silhouette of older bulls disproportionately, the dewlap acts as an indicator of age-related traits such as fighting experience. Facemask darkness, frontal hairbrush size and body greyness aligned with a third underlying variable, presumed to be androgen-related aggression. A longitudinal study provided independent support of these findings.

**Conclusion:**

The results show that the multiple agonistic signals in eland reflect three separate components of fighting ability: (1) body size, (2) age and (3) presumably androgen-related aggression, which is reflected in three backup signals. The study highlights how complex agonistic signalling systems can evolve through the simultaneous action of several selective forces, each of which favours multiple signals. Specifically, loud knee-clicking is discovered to be an honest signal of body size, providing an exceptional example of the potential for non-vocal acoustic communication in mammals.

## Background

Rivals often use agonistic signals to broadcast their fighting ability and thereby settle conflicts without incurring the high costs associated with actual fighting [1]. However, why males within many species have evolved several signals for this purpose calls for an explanation: given the costs of producing and receiving signals, why use more than one? Compared with the substantial research efforts aimed at clarifying the evolution of multiple sexual signals [2-4], the cause of multiple signalling in agonistic communication has remained neglected. We here test the applicability of the hypotheses on multiple sexual signalling in the intra-sexual context by deciphering the elaborate agonistic signalling system in the world's largest antelope, the eland (*Tragelaphus oryx*).

A key distinction between multiple signalling systems relies on whether the separate signals provide redundant information or not [5]. So far the best supported explanation for inter-sexual multiple signalling is probably the 'multiple messages hypothesis', which maintains that each signal trait conveys non-redundant information about a distinct aspect of quality [3,6,7]. Still, there is also considerable evidence to suggest that multiple sexual signals in some cases provide redundant information about the same underlying quality. In this situation, signals may coexist because they allow more accurate assessment by the receiver ('backup-signals hypothesis') [8] or because a variable sensory environment selects for multiple signals with different transmission properties ('multiple sensory environments hypothesis') [9].

When multiple signals are used in agonistic encounters, which components of fighting ability may interest the opponent? Key factors associated with fighting success are large body size, fighting experience and aggressiveness [10,11]. There is a strong relationship between male fighting success and body size in many taxa, including ungulates [12,13]. Body size can be reflected in acoustic signals, with reliability assured by the fact that body size determines the dimensions of the sound-producing organ and hence the acoustic structure of the sound produced (for example, in ungulates [14], monkeys [15], birds [16] and amphibians [17]). Although body size often has an overriding effect on fighting ability, fighting success may also increase with age independently of body size, either due to enhanced fighting experience or greater willingness to escalate fights as reproductive value decreases [18,19]. In this case, signalling of age can be a selective advantage. Fighting ability may furthermore depend on aggressiveness, which is often linked to fluctuating physiological states [20,21]. Thus, high androgen levels are a main determinant of aggressiveness and have been related to dark melanin-based colour signals in several vertebrates [22].

**Figure 1:**
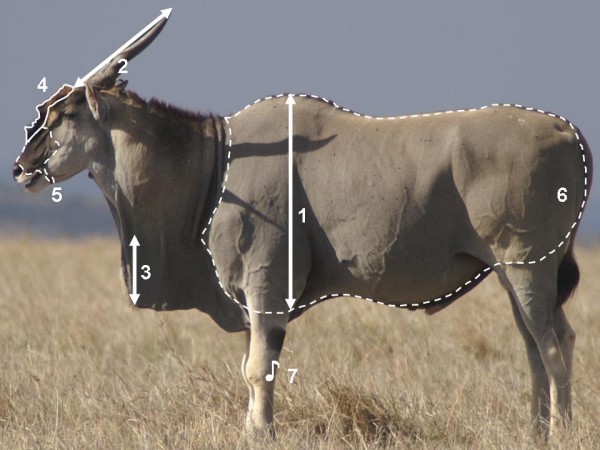
A lateral photo of a mature eland bull indicating the measures taken: (1) body depth, (2) horn length, (3) dewlap droop, (4) frontal brush size, (5) darkness of the facemask, (6) greyness of the body, and (7) knee-click frequency.

Focusing on eland bulls, we investigated the basis for coexistence of five signal traits, namely a pendant dewlap, a dark facemask, a frontal hairbrush, body greyness and a loud knee-clicking sound (Figure 1). During agonistic encounters, the four visual traits are presented in lateral display, which facilitates assessment by rivals [23,24], and here the dewlap can dramatically increase the silhouette of an opponent. With regards to dark facemasks in artiodactyls, as well as the often urine-soaked frontal hairbrush which is peculiar to eland bulls, support for signal functions has been found by [25] and [26] respectively. A communicative role has also been suggested for body colour, which ranges from reddish-brown to dark grey due to hair loss [24]. In addition to these visual traits, it has been hypothesized based on behavioural observations that the loud castanet-like click sound, which is emitted from the front knees of walking eland bulls, signals body size [26,27]. The sound, audible up to several hundred meters away [24], is likely to be costly by facilitating detection by predators, and the evolution of knee-clicking in the face of such costs indeed suggests a selective advantage in communication. For all these traits, however, their information content remains to be clarified, including how they interrelate and how they relate to age, body size and fluctuating aspects of condition.

In this study, we first used principal components analysis (PCA) to identify the independent factors underlying inter-individual variation in the signal traits. We predicted that if signal traits reflected the same quality, as proposed by the backup-signals and multiple sensory environments hypotheses, they would align with the same principal component. If, however, the signal traits reflected multiple messages, we predicted that they would align with different principal components, each reflecting a distinct aspect of fighting ability. In the analysis we also included proxy measures for age and body size, both of which are likely to affect fighting ability.

Moreover, we investigated the relationship between intra- and inter-individual trait variation in a longitudinal study, where we tested (a) whether traits were characterized by temporal fluctuations or directional change and (b) which traits co-varied within individuals over time. Correlated changes were predicted between signal traits and the component of fighting ability they reflected, as well as between traits signalling the same component of fighting ability.

On this basis, we here present simultaneous support for the multiple messages and backup signals hypotheses, and we specifically reveal the knee-clicking sound of mature eland bulls as an honest indicator of body size.

## Methods

### Study animal

Elands are nomadic browser-grazers forming unstable groups, which may be either unisexual or mixed [24,28]. The groups number up to several hundred individuals, although groups consisting exclusively of adults generally count less than 20 [29]. Elands are non-territorial and males are organized in a strong dominance hierarchy, which is believed to determine access to receptive females [30,31]. Dominance relations are settled mainly through agonistic signalling, fights being remarkably rare, even in the presence of oestrous females [24,26]. When they occur, fights are intense and take place by neck-wrestling, which renders body size, and in particular neck development, a decisive factor for the outcome. Fighting ability is also influenced by the fact that males in eland, in common with other tragelaphine antelopes, go through periodic states of heightened aggressiveness, so-called 'ukali' [27].

### Study area

The data were collected between 2006 and 2008 (February-May) from a 400 km^2 ^study area, partly situated within the Masai Mara National Reserve and the Olare Orok Conservancy, Kenya (1°20'S, 35°10'E). The landscape ranged from open grasslands to thickets of *Acacia drepanolobium *and *Croton dichogamus*, and the eland used both open and closed habitats. Individual eland were recognized from natural marks including the number and shape of vertical white body stripes, horn morphology, ear nicks and scars; in case of uncertainty, identity was established using a digital photo archive. Only mature bulls from the age of around four years old emit the knee-click sound [26], and the sound was chosen as the criterion for inclusion of individuals in the present study.

### Measurements of phenotypic traits

#### Visual traits

Using a method similar to [32], lateral photos of eland bulls standing relaxed were taken with a single-lens reflex digital camera (Konica Minolta Dynax 7D with a 400 mm lens) while the distance to the animal was measured simultaneously with a digital laser rangefinder (Bushnell Yardage Pro 800). A calibration curve for converting morphological measurements from pixels in photographic images to real-life metric measurements was obtained by taking photos of a 1 m pole at 1 m intervals and measuring its length in pixels. The measurements taken, which are indicated on Figure 1, were as follows: (1) body depth: the vertical distance from the highest point of the shoulders to the lowest point of the pectoral muscle, excluding any dewlap appended underneath; (2) horn length: the distance from the horn tip to the frontal insertion of the horn; (3) dewlap droop: the dewlap's maximum vertical droop from the neck; (4) frontal brush size: the two-dimensional area covered by the hairbrush on the forehead in profile; (5) facemask darkness: scored by an observer naïve to the study on a sliding scale from 1 (uniformly light) to 7 (black mask extending from the frontal brush to the chin); and (6) body greyness: scored by an observer naïve to the study on a sliding scale from 1 (reddish-brown) to 5 (dark grey). Body depth was included as a measure of body size, and horn length was used as a proxy for age, based on the fact that horn length of mature bulls decreases through life due to wear [26]. Body depth, horn length and dewlap droop were measured in DiMAGE Viewer version 2.37, while frontal brush size was measured in ImageJ version 1.38×. The morphometric measurements were based on an average of 4.6 ± 0.3 (mean ± SE) lateral photographs of each bull at each assessment, taken at varying distances, and repeatability, which was calculated following [33], was highly significant (*F*_61,172 _= 20.6, *P *< 0.001, repeatability 0.84, using body depth as an indicator).

#### Auditory trait

Knee-clicks were recorded at approximately 75 m distance using a solid state recorder (Marantz PMD670) with a directional microphone (Sennheiser ME67). Narrow-band spectrograms were generated in Praat version 4.3.20 (P Boersma and D Weenink, University of Amsterdam, The Netherlands) using the fast Fourier transform (FFT) method (window length = 0.03 s, time step = 1000, frequency step = 250, band width = 43 Hz, Gaussian window shape, dynamic range = 35 dB). The click sound is believed to be produced by standing waves in a knee-tendon, and supporting periodicity of the sound, the dominant frequency was generally clearly defined in the spectrograms. The dominant frequency, termed the knee-click frequency in the following, was measured in a power spectrum.

### Statistical analyses

In a cross-sectional study, we included data on the seven phenotypic traits from 48 adult males. Bartlett's test of sphericity showed the data set to be suitable for structure detection using PCA as variables were highly interrelated (χ^2 ^= 69.5, df 21, *P *< 0.001). Hence to identify the latent factors determining the relationships between the variables, we extracted the independent principal axes with eigen-values above unity using varimax rotation with Kaiser normalization.

In a longitudinal study, we included data on 14 adult males who were sampled at approximately one year's interval; we adjusted the changes in trait values to reflect 365 days exactly by dividing by the sampling interval. We then used *t*-tests to clarify whether there were (a) consistent directional changes in variables over time and/or (b) correlated changes in variables over time.

Assuming that the signal value of the metric signal traits is likely to reside in their size relative to body size (that is, change in proportions) rather than in their absolute size, the metric signal traits were controlled for body size by dividing with body depth for unidimensional traits or its quadrate for two-dimensional traits. However, all analyses were also done using absolute trait values, which yielded similar results in general; these results are therefore only shown where they differed from those obtained using relative values in whether they were significant or not (α = 0.05, two-sided). All statistical analyses were done in SPSS version 15.0.0 (SPSS, Chicago, IL).

## Results

### Inter-individual analysis

The mean ± SE (range) of the seven phenotypic traits were as follows: body depth 84.7 ± 0.34 (79.8–89.9) cm, horn length 53.8 ± 0.86 (44.1–66.6) cm, dewlap droop 24.9 ± 0.54 (17.2–32.4) cm, frontal brush size 56.6 ± 4.33 (2.5–116.0) cm^2^, knee-click frequency 3060 ± 26.8 (2776–3580) Hz, facemask darkness score 3.7 ± 0.25 (1–7), and body greyness score 1.6 ± 0.14 (1–5).

**Figure 2:**
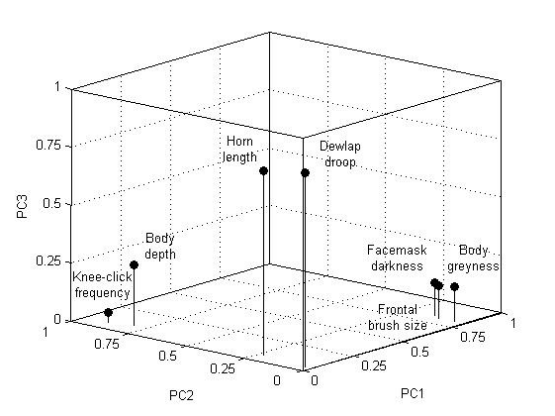
**The traits plotted against the three principal components using their numeric Pearson correlation coefficients**.

**Figure 3:**
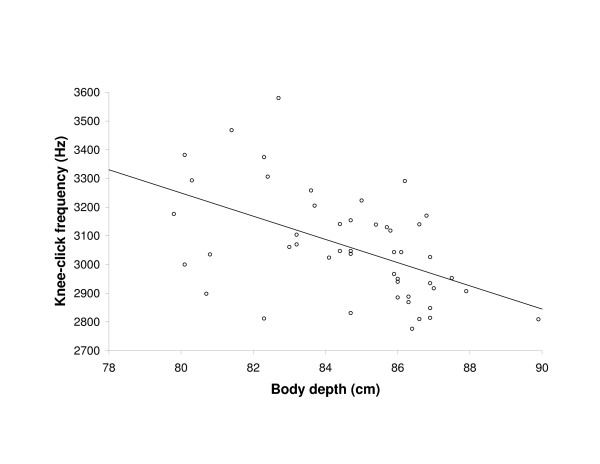
**The dominant frequency of knee-clicks in relation to body depth.** Linear regression line shown (*P *< 0.001).

The PCA identified three underlying principal components, which divided the traits analyzed into three distinct groups (Table 1; Figure 2). Frontal brush size, facemask darkness and body greyness all showed a significant positive correlation with the first principal component (PC1), as well as with each other, in bivariate analyses. Body depth and knee-click frequency correlated with the second principal component (PC2), positively and negatively respectively, and they also correlated negatively with each other (Figure 3; Additional files 1 and 2). Finally, horn length, the proxy measure of age, and dewlap droop correlated with the third principal component (PC3), negatively and positively respectively, and they also correlated negatively with each other. Body depth, which was negatively correlated with horn length, only correlated with dewlap droop when using absolute measures, the relationship in this case being positive (Pearson correlation: *r *= 0.353, *N *= 48, *P *= 0.014).

**Table 1:** Pearson correlations between principal components and phenotypic traits

	PC1 (eigen-value 2.04)	PC2 (eigen-value 1.55)	PC3 (eigen-value 1.45)	Facemask darkness	Frontal brush size	Body depth	Knee-click frequency	Horn length	Dewlap droop
Body greyness	0.816***	-0.044	0.148	0.514***	0.503***	0.162	0.017	-0.199	0.053
Facemask darkness	0.835***	0.146	-0.139		0.543***	0.127	-0.196	0.026	0.027
Frontal brush size	0.809***	0.109	0.140			0.140	-0.181	-0.175	0.145
Body depth	0.091	0.805***	0.260				-0.515***	-0.304*	0.183
Knee-click frequency	-0.070	-0.902***	0.042					-0.186	-0.012
Horn length	-0.075	-0.233	-0.792***						-0.387**
Dewlap droop	0.043	-0.028	0.833***						

The analysis explained 72% of the variance in the variables. * *P *< 0.05, ** *P *< 0.01, *** *P *< 0.001.

### Intra-individual analysis

**Figure 4:**
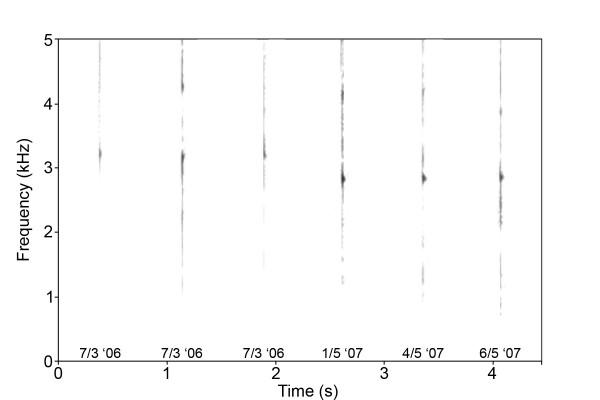
**Narrow-band spectrograms (window length = 0.03 s, band width = 43 Hz, Gaussian window shape) of knee-clicks from the same bull recorded in two consecutive years**. Note the drop in knee-click frequency between years compared with the constancy over the short term.

In the longitudinal study, no significant directional trend could be demonstrated for any of the traits related to PC1 (frontal brush size: *t *= 0.765, df 13, *P *= 0.458; facemask darkness: *t *= 0.186, df 13, *P *= 0.856; body greyness: *t *= -1.203, df 13, *P *= 0.250). In contrast, all the traits related to PC2 and PC3 showed significant temporal trends, either increasing (estimated annual changes: body depth 1.92 ± 0.58 cm (mean ± SE), *t *= 3.33, df 13, *P *= 0.005, dewlap droop 1.98 ± 0.55 cm, *t *= 2.33, df 13, *P *= 0.037) or decreasing (estimated annual changes: horn length -1.35 ± 0.31 cm, *t *= -4.37, df 13, *P *= 0.001, knee-click frequency -160 ± 46 Hz, *t *= -3.45, df 13, *P *= 0.004; Figure 4).

**Figure 5:**
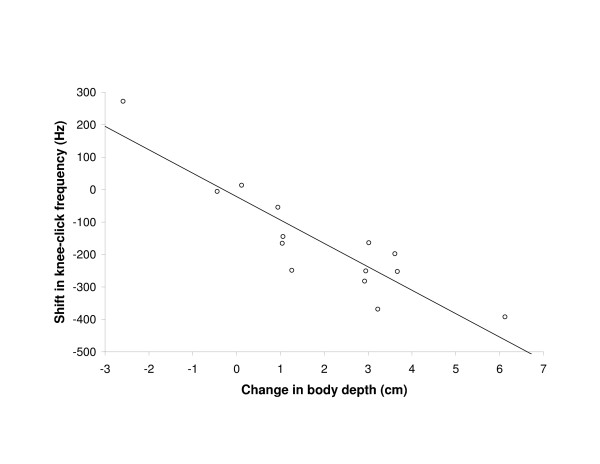
**The shift after one year in the dominant frequency of the knee-click sound of individual bulls in relation to their change in body depth.** Linear regression line shown (*P *< 0.001).

Bivariate comparisons revealed significantly correlated changes between all the PC1-related variables (Pearson correlation: body greyness/facemask darkness *r *= 0.608, *N *= 14, *P *= 0.021; body greyness/frontal brush size *r *= 0.592, *N *= 14, *P *= 0.026; facemask darkness/frontal brush size *r *= 0.671, *N *= 14, *P *= 0.009) as well as between body depth, knee-click frequency and facemask darkness (Pearson correlation: body depth/knee-click frequency *r *= -0.899, *N *= 14, *P *< 0.001, Figure 5; body depth/facemask darkness *r *= 0.583, *N *= 14, *P *= 0.029; knee-click frequency/facemask darkness: *r *= -0.598, *N *= 14, *P *= 0.024).

## Discussion

We here show that the dominant frequency of the knee-clicks in eland is an honest signal of body size, reflecting both inter-individual variation and intra-individual changes over time. The dewlap droop increased with age (as estimated from horn length) rather than with body size, and thus appears as an indicator of age-related traits, such as fighting experience. Finally, facemask darkness, frontal brush size and body greyness reflected yet another underlying variable, presumed to be androgen-related aggressiveness due to its association with hair darkness. These results support the multiple messages hypothesis, with three distinct components of fighting ability reflected in the signal traits (see also [34]). However, in simultaneous support of the backup-signals hypothesis, the component presumed to be androgen-related aggressiveness was represented by three signal traits.

The discovery that knee-clicks in eland honestly indicate body size reveals an unusual potential for non-vocal acoustic communication in mammals. It is believed that the click is produced when a tendon slips over a carpal bone [24], and such a mechanism would explain why the dominant frequency correlates negatively with body size. The tendon in this case behaves like a string being plucked, and the frequency of a standing wave in a string correlates negatively with both its length and diameter. Thus, most importantly, the length of the tendon is predicted to increase with skeletal measures, which generally increase with age. In addition, the diameter of the string is predicted to increase with muscle mass, and tellingly, the one case in the present study where click frequency clearly increased, rather than decreased, over time coincided with a considerable decrease in body depth due to visible loss of body condition and atrophy of the pectoral muscle. Hence, in common with ungulate vocal communication [14], physical constraints appear to assure that eland knee-clicking honestly reflects body size, in this case incorporating both skeletal measures and muscle mass. Eland bulls are mute compared with their relatives [24,26], and knee-clicking is possibly favoured over vocal signals as a cheaper option for sustained acoustic status signalling in the unstable multi-male herds. Similar click sounds are produced by another gregarious ungulate, the reindeer (*Rangifer tarandus*) [35]; however, the function of the clicks in this species remains to be investigated.

From its association with PC3, which reflected estimated age, the dewlap appears as a reliable indicator of age-related traits such as fighting experience. Bulls may also benefit from signalling advanced age if older individuals, having lower reproductive value, incur lower costs from injury and therefore are more risk-prone and dangerous adversaries [19]. Finally, it could be significant that the dewlap enhances the silhouette presented in lateral displays, particularly exaggerating the size of the neck which is crucial in the neck-wrestling combats [24]: older males may in this way bluff their opponents, even masking muscle loss once they are past their prime.

With regards to PC1, the component's association with facemask darkness suggests that in this case androgen-related aggressiveness may be the underlying aspect of fighting ability being signalled. Androgens have been linked to dark melanin pigmentation of hair in several mammalian species, for example, white-tailed deer pelage (*Odocoileus virginianus) *[36], lion manes (*Panthera leo) *[37], and coat colour in mice (*Mus musculus) *[38]). The PC1-related traits contrasted with the PC2- and PC3-related traits in that we did not detect any strong directional trend within individuals over time, a finding which is indeed consistent with PC1 reflecting a fluctuating endocrine state such as androgen levels. These results highlight the possibility that the PC1-related traits signal the aforementioned 'ukali' state of periodic aggressiveness [27], which is likely to be caused by temporary endocrine changes. Finally, the positive effect of androgens on growth of somatic tissue [39] can explain why the intra-individual changes in facemask darkness and body depth were positively correlated.

## Conclusion

Three determinants of fighting ability are broadcasted in the agonistic communication of eland bulls, that is, body size, age and presumably androgen-related aggression, the latter reflected in three redundant signals. These findings provide simultaneous support for the multiple messages and backup signals-hypotheses of multiple signalling, and the study thus not only identifies commonalities between intra- and inter-sexual multiple signalling, but also points to the possibility that complex multiple signalling systems often originate from the concerted action of several selective forces, each of which favours multiple signals. In addition, the signal value of the peculiar knee-clicking sound, which reliably indicates body size, provides an intriguing example of how information may be transferred through non-vocal acoustic communication in mammals.

## Authors' contributions

JBJ conceived the project, collected and analyzed the data, and drafted the manuscript. TD contributed to data acquisition and revised the manuscript.

## Supplementary Material

Additional file 1 **A walking eland bull knee-clicking.** Click here for file

Additional file 2 **Knee-clicks from a walking eland bull.** Click here for file

## References

[B1] Searcy WA, Nowicki S (2005). The Evolution of Animal Communication.

[B2] Bradbury JW, Vehrencamp SL (1998). Principles of Animal Communication.

[B3] Candolin U (2003). The use of multiple cues in mate choice. Biol Rev.

[B4] van Doorn GS, Weissing FJ (2006). Sexual conflict and the evolution of female preferences for indicators of male quality. Am Nat.

[B5] Partan SR, Marler P (2005). Issues in the classification of multimodal communication signals. Am Nat.

[B6] Møller AP, Pomiankowski A (1993). Why have birds got multiple sexual ornaments?. Behav Ecol Sociobiol.

[B7] Johnstone RA (1995). Honest advertisement of multiple qualities using multiple signals. J Theor Biol.

[B8] Johnstone RA (1996). Multiple displays in animal communication: 'backup signals' and 'multiple messages'. Phil Trans R Soc London B.

[B9] Reynolds JD (1993). Should attractive individuals court more? Theory and a test. Am Nat.

[B10] Jarman PJ (1983). Mating systems and sexual dimorphism in large, terrestrial, mammalian herbivores. Biol Rev.

[B11] Andersson M (1994). Sexual Selection.

[B12] Clutton-Brock TH, Guinness FE, Albon SD (1982). Red deer – behaviour and ecology of two sexes.

[B13] Pelletier F, Festa-Bianchet M (2006). Sexual selection and social rank in bighorn rams. Anim Behav.

[B14] Reby D, McComb K (2003). Anatomical constraints generate honesty: acoustic cues to age and weight in the roars of red deer stags. Anim Behav.

[B15] Ghazanfar AA, Turesson HK, Maier JX, van Dinther R, Patterson RD, Logothetis NK (2007). Vocal-tract resonances as indexical cues in rhesus monkeys. Curr Biol.

[B16] Ryan MJ, Brenowitz EA (1985). The role of body size, phylogeny, and ambient noise in the evolution of bird song. Am Nat.

[B17] Davies NB, Halliday TR (1978). Deep croaks and fighting assessment in toads *Bufo bufo*. Nature.

[B18] Rutberg AT (1986). Dominance and its fitness consequences and American bison cows. Behaviour.

[B19] Parker GA (1974). Assessment strategy and the evolution of fighting behavior. J Theor Biol.

[B20] Svare BB (1983). Hormones and Aggressive Behavior.

[B21] Hau M (2007). Regulation of male traits by testosterone: implications for the evolution of vertebrate life histories. BioEssays.

[B22] Ducrest A-L, Keller L, Roulin A (2008). Pleiotropy in the melanocortin system, coloration and behavioural syndromes. Trends Ecol Evol.

[B23] Clutton-Brock TH, Albon SD (1979). The roaring of red deer and the evolution of honest advertisement. Behaviour.

[B24] Estes RD (1991). The Behavior Guide to African Mammals.

[B25] Stoner CJ, Caro TM, Graham CM (2003). Ecological and behavioral correlates of coloration in artiodactyls: systematic analyses of conventional hypotheses. Behav Ecol.

[B26] Hillman JC (1979). The biology of the eland (*Taurotragus oryx *Pallas) in the wild. PhD thesis.

[B27] Kingdon J (1982). East African mammals.

[B28] Hofmann RR, Stewart DRM (1972). Grazer or browser: a classification based on stomach structure and feeding habits of east African ruminants. Mammalia.

[B29] Hillman JC (1987). Group size and association patterns in the common eland *Tragelaphus oryx*. J Zool.

[B30] Gosling LM, Rubenstein DI, Wrangham RW (1986). The evolution of mating strategies in male antelopes. Ecological Aspects of Social Evolution.

[B31] Clutton-Brock TH (1989). Mammalian mating systems. Proc R Soc London B.

[B32] Bro-Jørgensen J, Durant SM (2003). Mating strategies of topi bulls: getting in the centre of attention. Anim Behav.

[B33] Lessells CM, Boag PT (1987). Unrepeatable repeatabilities: a common mistake. Auk.

[B34] Stuart-Fox DM, Firth D, Moussalli A, Whiting MJ (2006). Multiple signals in chameleon contests: designing and analysing animal contests as a tournament. Anim Behav.

[B35] Banfield AWF, Smith IN (1966). The caribou. The Unbelievable Land.

[B36] Bubenik GA, Bubenik AB (1985). Seasonal variations in hair pigmentation of white-tailed deer and their relationship to sexual activity and plasma testosterone. J Exp Zool.

[B37] West PM, Packer C (2002). Sexual selection, temperature, and the lion's mane. Science.

[B38] Wierzbicki H (2000). Effect of melanin biosynthesis on the coat colour of animals. Med Weter.

[B39] Eckert R, Randall D, Augustine G (1988). Animal Physiology – Mechanisms and Adaptations.

